# Current status and future directions of application of urine proteomics in neonatology

**DOI:** 10.3389/fped.2024.1509468

**Published:** 2025-01-14

**Authors:** Dan Wu, Lulu Zhang, Fangrui Ding

**Affiliations:** ^1^ Tianjin Institute of Obstetrics and Gynecology, Tianjin Central Hospital of Obstetrics and Gynecology, Tianjin, China; ^2^ Tianjin Key Laboratory of Human Development and Reproductive Regulation, Tianjin Central Hospital of Obstetrics and Gynecology, Tianjin, China; ^3^ Department of Neonatology, Tianjin Central Hospital of Obstetrics and Gynecology, Tianjin, China

**Keywords:** urinary proteomics, neonatology, biomarker discovery, mass spectrometry, bioinformatics

## Abstract

With continuous advancements in mass spectrometry technology, researchers increasingly utilize this method to investigate the molecular mechanisms underlying various diseases, and to identify novel diagnostic and therapeutic strategies. Among proteomics applications, urinary proteomics stands out for its non-invasive nature, making it particularly suitable for vulnerable populations like neonates. This review provides a comprehensive overview of recent research on urinary proteomics in the field of neonatology. It summarizes findings from numerous studies, illustrating how urinary proteomic profiles provide critical insights into neonatal health and disease. By identifying specific protein biomarkers in urine, researchers can gain insights into the early detection and monitoring of neonatal diseases, potentially leading to more timely and effective interventions. As technology evolves, the sensitivity and accuracy of proteomic analyses are expected to improve, opening new avenues for research and clinical applications.

## Background

Since the advent of mass spectrometry, researchers have revolutionized disease research by applying proteomics to uncover biomarkers and predictive models. By integrating bioinformatics, machine learning and other technologies, numerous biomarkers for disease diagnosis have been identified, and a lot of predictive models have been developed, particularly for life-threatening diseases such as cancer and diabetic nephropathy (1–6). Under normal physiological conditions, 70% of urinary proteins originate from the kidneys, while the remaining proteins come from peptides and small proteins that are filtered from the blood or secreted into the urine (7). Therefore, urinary proteomics can effectively reflect the health status of the kidneys and other organs (8).

Using proteomics for neonatal disease research has several advantages. First, urine, as a bodily fluid that can be produced in large quantities, is non-invasive and does not cause any pain or discomfort to neonates, making it suitable for frequent sampling and dynamic monitoring of neonatal health. This makes it an excellent source of biomarkers. Second, the process of collecting urine samples is simple and easy to implement in clinical settings, facilitating large-scale screening.

In the field of neonatology, due to their vulnerability, some invasive procedures are challenging to apply, or the application of invasive procedures could pose significant harm to newborns. For instance, current studies revealed that blood sampling from newborns can result in more than 10% loss of blood volume (9, 10). Therefore, the exploration and identification of non-invasive testing methods hold substantial promise. Urinary proteomics is emerging as a pivotal methodology in this domain.

**Figure 1:**
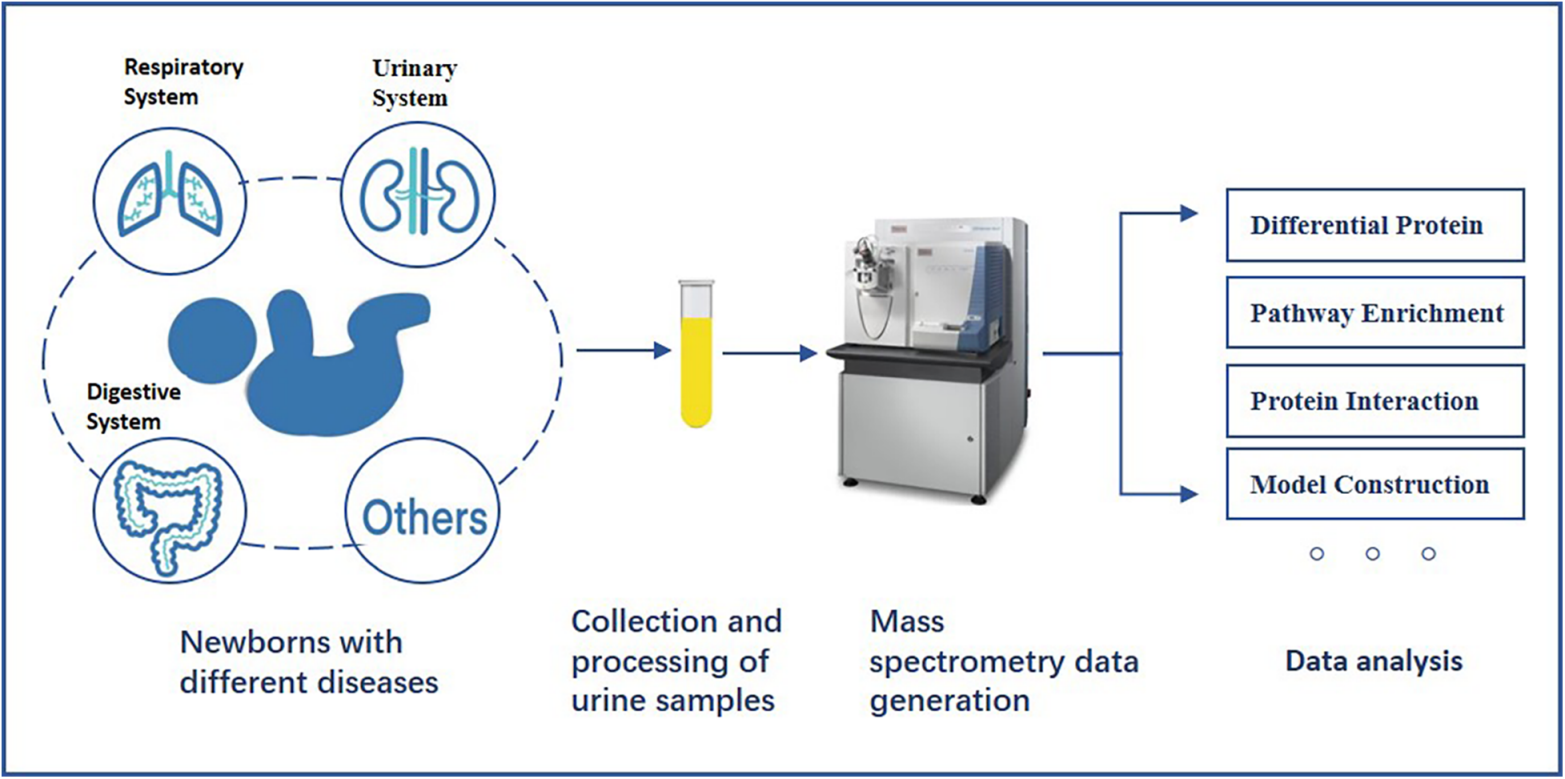
The general process of studying the proteome of newborn urine.

Several studies have explored the use of urinary proteomics to investigate molecular mechanisms and aid in disease diagnosis in neonatal diseases. However, the clinical application and value of urinary proteomics remain limited. The purpose of this review is to summarize the findings of urinary proteomics research in neonatal diseases, with a primary focus on kidney injury, respiratory system underdevelopment, necrotizing enterocolitis and so on. We will discuss the clinical applications and potential of these studies in predicting neonatal diseases, providing valuable reference points for future diagnosis and treatment. Given its non-invasive nature and easy accessibility, urine has the potential to become a new diagnostic tool for both neonatal and adult diseases, especially neonatal kidney diseases. Finally, we explore the prospects of integrating urinary proteomics with multi-omics and artificial intelligence, offering new insights for medical practice and industrial applications. Figure 1 provides an overview of research in the proteome of newborn urine.

This review summarizes the potential role of urinary proteomics in predicting neonatal diseases. We conducted a database search on PUBMED and WEB OF SCIENCE using keywords such as “neonatal”, “infant”, “premature”, and “urinary proteomics”, and reviewed the references in related articles. All the studies included are from English-language publications and focus on urinary proteomics research in neonates. Figure 1 illustrates the current landscape of neonatal urinary proteomics research, while Table 1 provides a detailed summary of studies included in this review, highlighting their key findings and methodologies.

**Table 1:** Summary of proteomic research on neonatal urine.

Author	Publication year	Disease	Methods	Sample size	Results	Mentioned biomarkers
Stephane Decramer et al. (11)	2006	Ureteropelvic Junction Obstruction (UPJO)	CE-MS	71 (20 from healthy adults, 13 from healthy newborns, 19 from No_OP individuals, 19 from OP individuals)	They identified 51 peptides distinguishing health from UPJO. Validation showed 94% sensitivity, 80% specificity for No_OP, and 100% for OP. Predictions for surgery candidates: 25 progressed to OP, 11 to No_OP. The study indicated 94% accuracy for surgery prediction, aiding early intervention in UPJO infants, and potentially reducing invasive procedures.	–
Jens Drube et al. (12)	2010	Ureteropelvic Junction Obstruction (UPJO)	CE-MS	27 (23 with UPJO, 4 without UPJO)	In 19 infants under one year of age, the sensitivity for predicting UPJO was 83%, with a specificity of 92%. However, as children aged, sensitivity decreased to 20% and specificity to 66%. Newborn urine is more suitable than that of older children for predicting UPJO progression.	–
John W. Froehlich et al. (13)	2016	Ureteropelvic Junction Obstruction (UPJO)	CE-MS	16 (8 with UPJO, 8 without UPJO)	They identified over 1,113 proteins and found 74 differentially expressed proteins between the groups. These proteins are enriched in pathways related to oxidative stress/ROS processing, fibrosis, and acute-phase inflammation.	–
Young Hwa Jung et al. (14)	2020	Acute Kidney Injury (AKI)	LC-MS/MS	35 (5 for discovery, 16 for verification, 14 for validation)	Six proteins were identified as biomarkers for predicting AKI. A multifactorial logistic regression model was constructed using their expression levels in conjunction with NGAL, Annexin A5, and Protein S100-P, achieving an AUC of 0.932, indicating significant clinical relevance.	NGAL, CILP-2, 6-PGLS, annexin A5, galectin 3, protein S100-P
Natalia L. Starodubtseva et al. (15)	2015	Congenital Pneumonia	LC-MS/MS	32 (27 preterm newborns, 5 full-term newborns)	434 proteins and 1,888 peptides were identified, and the differential proteins were related to the inflammatory process.	–
Natalia L. Starodubtseva et al. (16)	2016	Respiratory Diseases	LC-MS/MS	37 (27 preterm newborns, 10 full-term newborns)	813 proteins and 3,672 peptides were identified. Perform differential protein analysis using bioinformatics methods and annotate differential proteins using GO database, enriched in cell adhesion, enzyme activity regulation, inflammation, and stress response processes.	–
Ahmed Saima et al. (17)	2022	Bronchopulmonary Dysplasia (BPD)	LC-MS	42 (21 newborns with BPD, 21 without BPD)	They identified 16 proteins with significant abundance differences that are targets of FDA-approved drugs, six of which are previously associated with BPD development.	P13796, Q9Y3B3, P11142, P08238, P14384, P15085, P05534
Karl G. Sylvester et al. (18)	2014	Necrotizing Enterocolitis (NEC)	LC-MS	119 (85 newborns with NEC, 17 with sepsis, 17 controls)	Seven biomarkers were identified. A genetic algorithm model of these biomarkers predicted NEC and sepsis in the validation group with an AUC of 98.2, and medical NEC vs. surgical NEC with an AUC of 98.4.	A2ML1, CD14, CST3, FGA, PEDF, RET4, VASN
Stephen Mackay et al. (19)	2024	Necrotizing Enterocolitis (NEC)	Aptamer-based protein microarray	40 (20 newborns with NEC, 8 age-matched, 12 self-matched)	Two panels were built to differentiate between infants with and without NEC.	REG1B, REG3A, FABP2, DEFA5; REG1B, SSBP1, CRYZL1, ITM2B, IL36B, IL36RN
Estela Cabral et al. (20)	2017	Cardiovascular Disease	LC–MS/MS	8 (4 preterm newborns, 4 full-term newborns)	Researchers believe that the metabolic adaptation and cardiac growth pathways enriched by differential proteins may be the reasons for the increased risk of cardiovascular disease in premature infants.	–
Claudia Abeijon et al. (21)	2019	Visceral Leishmaniasis (VL)	Mass Spectroscopy	95 (55 newborns with VL, 40 healthy controls)	Specific proteins were identified in urine and five biomarkers were screened for the diagnosis of visceral leishmaniasis, with a sensitivity of 82.2%. This study contributes to the predictive diagnosis of neonatal leishmaniasis.	Li-isd1, Li-txn1, and Li-ntf; Ld-mao1 and Ld-ppi1
Sumrati Gurtoo et al. (22)	2023	Hypoxic-ischemic Encephalopathy	LC–MS/MS	38	Biomarker proteins are linked to pathways including amyloid fiber formation, programmed cell death diseases, reactive oxygen species detoxification, and neurodegenerative diseases.	APOD, ORM1, SOD1, ABP1
Maire Brasseler et al. (23)	2024	Gestational Age	LC-MS	51 (24 preterm newborns, 21 full-term newborns)	Peptides in the urine of full-term and preterm infants were identified using mass spectrometry, and peptides were associated with gestational age and clinical risk scores. Using the identified proteins, it is possible to identify newborns with poor clinical outcomes in the future.	SLC38A10

## Urinary system diseases

The kidneys produce urine through the filtration process, which is mainly used to excrete soluble waste such as electrolytes, hormones, nitrogen-containing compounds, etc. (24). The glomerulus filters approximately 150–180 liters of plasma to form original urine, with over 99% of the original urine being reabsorbed by the renal tubules and ultimately excreted through the urethra. The normal daily urine output should be less than 150 mg/L, with 30% of the protein originating from plasma and 70% coming from the kidneys (7). Therefore, using urine proteomics to identify renal injury in newborns is an effective approach. In fact, according to our search results, urinary proteomics has been the most extensively studied for neonatal kidney injury and other urinary system related diseases.

Ureteropelvic Junction Obstruction (UPJO) is a common neonatal kidney disease that can lead to renal developmental abnormalities and loss of renal function. Early identification of UPJO severity and the need for surgery is clinically significant. In 2006, Stephane et al. used capillary electrophoresis-mass spectrometry (CE-MS) to analyze peptide patterns in the urine of 103 UPJO newborns, comparing them to 13 healthy newborns and 20 adults, respectively. They found that the urinary distribution in newborns was more consistent compared to adults, which allowed a better definition of pathological peptides. Newborns were divided into three groups: undiagnosed UPJO (No_OP), potential surgery candidates (OP_Poss), and severe UPJO requiring immediate surgery (OP). Analysis of biomarkers between healthy, No_OP, and OP groups revealed 51 distinct peptides. An independent validation set showed a classification sensitivity of 94%, with No_OP specificity of 80% and OP specificity of 100%. Prospective predictions for 36 infants in the OP_Poss group indicated that 25 would progress to OP and 11 to No_OP. Nine months later, 13 infants recovered spontaneously, and 23 underwent surgery, yielding a prediction accuracy of 94% (11). This study suggests that urine proteomics analysis can help predict clinical outcomes in congenital UPJO, enabling early intervention and potentially protecting future kidney function while reducing the need for invasive procedures in newborns.

In 2010, Jens et al. extended Stephane's research (11) using CE-MS to analyze the proteome of 27 children, 11 of whom had UPJO. They used the previously identified 51 peptides to predict the need for surgery in UPJO patients. Among 19 newborns under one year, the sensitivity and specificity were 83% and 92%, respectively. However, with increasing age, sensitivity dropped to 20% and specificity to 66% (12). This aligns with Stephane's findings that the urine proteome of newborns better predicts UPJO progression than that of older children (11), highlighting the clinical significance of proteomics in diagnosing surgical needs in UPJO newborns. Hrair et al. conducted a cost analysis of Stephane's findings to assess the method's suitability for widespread clinical use. Their preliminary evaluation suggests that this approach could reduce healthcare costs and improve the quality-adjusted life years (QALYs) of UPJO patients (25, 26).

The study conducted by Stephane Decramer in 2006 has significant implications for predicting the progression of UPJO in newborns (11). Their research demonstrated the utility of using peptides to classify newborns who required surgery for UPJO from those who did not, providing a potentially valuable tool for clinical decision-making. However, the study focused primarily on the classification aspect and did not fully explore the biological significance of these peptides. This limitation highlights the need for further research to investigate the biological pathways and molecular mechanisms underlying these differentially expressed peptides. Understanding the physiological roles and interactions could provide deeper insights into the pathogenesis of UPJO, potentially leading to more targeted and effective therapeutic strategies.

In 2016, John et al. used CE-MS to study the urinary proteomics of 8 newborns with UPJO and 8 healthy newborns, identifying proteins and related biological networks involved in UPJO. They identified over 1,113 proteins and found 74 differentially expressed proteins between the groups. These proteins are enriched in pathways related to oxidative stress/ROS processing, fibrosis, and acute-phase inflammation (13). This study enhances the understanding of proteomic changes in UPJO and its biological mechanisms, contributing significantly to basic scientific research on UPJO.

Acute kidney injury (AKI) is common in premature infants and poses significant health risks. Existing biomarkers for AKI lack predictive performance and timely detection capabilities. In 2020, Jung et al. studied five neonates with AKI before and after being treated with ibuprofen, they detected 1,810 proteins using LC-MS/MS. They focused on 174 proteins that were upregulated or downregulated, identifying six candidate biomarkers: NGAL, CILP-2, 6-PGLS, annexin A5, galectin 3, and protein S100-P. For validation, they analyzed urine samples from 16 AKI and 16 healthy newborns using DIA-MS, quantifying 168 proteins and finding significant differences in six. ELISA analysis of 14 AKI and 14 healthy newborns showed that NGAL, annexin A5, and protein S100-P had sensitivities and specificities greater than 0.7. Combining these three biomarkers with logistic regression yielded an AUC of 0.932, indicating a high predictive value (14). This study, involving LC-MS/MS, DIA-MS, and ELISA methods, systematically screened and validated AKI-related proteins, demonstrating robust experimental design and significant clinical application value.

The kidneys are the primary source of proteins in urine, making neonatal urinary proteomics a valuable tool for studying kidney development, particularly in premature infants (27, 28). Non-invasive urine collection allows for painless sampling and provides an effective way to monitor renal development over time, which is crucial for clinical decision-making in neonatal care. Despite its potential, research in this area remains limited. Future studies should focus on exploring neonatal urine proteins to better understand growth and development, and identify novel biomarkers for clinical applications.

## Respiratory disease

The respiratory system diseases are often accompanied by systemic physiological changes. Disease-related proteins may circulate through the bloodstream, be filtered by the kidneys, and appear in urine. In recent years, some researchers have begun using urinary proteomics to identify biomarkers for neonatal respiratory system diseases.

In 2015, Natalia et al. collected urine samples from 32 newborns in the neonatal intensive care unit, including 27 premature infants, 4 extremely low birth weight infants, 7 low birth weight infants, and 17 cases of congenital pneumonia. Using LC-MS/MS, they identified 434 proteins and 1,888 peptides. Principal component analysis revealed differences in urinary protein levels between newborns with congenital pneumonia and those with non-infectious respiratory diseases. Differentially expressed proteins were linked to the inflammatory process, with α2-antiplasmin and α1-antitrypsin identified as reliable markers of acute inflammation. This study suggests that urinary proteomics can be used for the prediction and non-invasive diagnosis of respiratory diseases in newborns (15). In a subsequent study conducted in 2016, Natalia et al. studied the urine proteome of 37 premature infants with respiratory diseases and 10 full-term newborns within 7 days of birth, identifying 813 proteins and 3,672 peptides. Bioinformatics analysis and GO database annotation revealed proteins involved in cell adhesion, enzyme activity regulation, inflammation, and stress responses. This study further demonstrates the potential of neonatal urinary proteomics in diagnosing and predicting respiratory diseases in newborns (16).

In 2022, Ahmed et al. identified biomarkers for bronchopulmonary dysplasia (BPD) in neonatal urine proteomics using LC-MS. BPD is a chronic lung disease affecting premature infants, often developing from respiratory distress syndrome. Early identification of infants at risk for BPD is crucial for timely treatment. The study analyzed urine samples from 21 premature and 21 healthy infants, identifying 35 significantly upregulated proteins linked to immune processes, leukocyte-mediated immunity, and complement activation. Pathway analysis of 57 significantly downregulated proteins revealed associations with neutrophil degranulation, leukocyte activation. They identified 16 proteins with significant abundance differences that are targets of FDA-approved drugs, six of which are previously associated with BPD development. These findings offer insights for drug target screening in BPD (17).

In respiratory disease, researchers have focused on applying bioinformatics methods to analyze the biological mechanisms of newborn urine. The results show that the pathways related to the respiratory system primarily involve inflammation and immune responses (15–17). In the future, incorporating more samples of relevant studies may enable the construction of predictive models for neonatal respiratory system diseases.

## Digestive system diseases

Inflammatory response in the digestive system can also be reflected in the proteome of newborn urine. Colorectal inflammation is the most extensively studied area in this field.

In 2014, Karl et al. collected urine samples from 119 premature infants [85 with necrotizing enterocolitis (NEC), 17 with sepsis, and 17 controls]. LC/MS was used to identify candidate biomarkers in 59 samples, and enzyme-linked immunosorbent assay (ELISA) validated these biomarkers in the remaining 60 samples. Seven biomarkers were identified: alpha-2 macroglobulin-like protein 1, CD14, cystatin 3, fibrinogen alpha chain, pigment epithelium-derived factor, retinol-binding protein 4, and vasolin. ELISA confirmed these findings. A genetic algorithm model of these biomarkers predicted NEC and sepsis in the validation group with an AUC of 98.2, and medication vs. surgical NEC with an AUC of 98.4. This approach enhances early NEC diagnosis and identifies those at risk for severe disease (18).

In 2024, Stephen et al. studied urinary proteomics related to NEC using aptamer-based proteomics on 20 NEC newborns and 20 controls. They identified 7,596 proteins, 99 proteins shared between the two groups, and 150 proteins showed significant differences, potentially serving as NEC biomarkers. Two panels were constructed: Panel 1, for identifying NEC and intestinal epithelial hyperplasia (REG1B, REG3A, FABP2, DEFA5), had an AUC of 0.90. Panel 2, for distinguishing NEC from controls (REG1B, SSBP1, CRYZL1, ITM2B, IL36B, IL36RN), had an AUC of 0.98. This study provides a basis for the early diagnosis of neonatal necrotizing enterocolitis (19).

However, only a small number of studies have studied NEC from the perspective of urine proteomics, and some studies collect fecal samples of NEC infants for proteomic analysis (29). More research is needed to compare which method is more effective.

## Other diseases

In addition to the several types of diseases mentioned above, there are also many neonatal diseases studied using urine proteomics methods.

Premature birth is associated with increased cardiovascular disease (CVD) risk in adulthood. In 2017, Estela et al. used mass spectrometry to analyze urine proteins from premature (*n* = 4) and full-term newborns (*n* = 4). They identified 434 proteins, with 126 common to both groups, 37 specific to premature infants, and 38 specific to full-term newborns. Differential protein analysis revealed enrichment in immune system and metabolic pathways in premature infant urine. These pathways, involving metabolic adaptation and cardiac growth, may contribute to the increased CVD risk observed in premature infants. The study suggests potential biomarkers in neonatal urine for predicting adult CVD risk, yet validation in adult samples is needed for clinical application (20).

Visceral leishmaniasis (VL) is a serious parasitic disease. In 2019, Claudia et al. conducted a multicenter study and collected urine samples from 95 newborns in Brazil and Kenya, including 40 samples from healthy newborns, 55 samples from newborns with VL, and specific proteins were identified in their urine. Their study identified and validated two L. donovani proteins as diagnostic biomarkers for VL, which are maoc family dehydratase-like protein (Ld-mao1) and peptidyl-prolyl cis-trans isomerase/rotamase (Ld-ppi1). These findings contribute to the development of predictive diagnostic methods for neonatal leishmaniasis (21).

In 2023, Sumrati et al. conducted a case-control study involving 38 newborns to investigate ischemic encephalopathy (HIE), a serious birth complication. Serum and urine samples collected 24 h after birth were analyzed using protein mass spectrometry, identifying promising biomarkers such as APOD, ORM1, SOD1, and FABP1. These proteins are linked to pathways including amyloid fiber formation, programmed cell death diseases, reactive oxygen species detoxification, and neurodegenerative diseases. This study aims to identify specific biomarkers, for screening neonates at risk of brain injury, facilitating early detection and precise monitoring of injury progression. Early and accurate biomarker identification is crucial for reducing mortality rates and long-term neurodevelopmental impairments in affected neonates. This research enhances understanding of molecular indicators of neonatal brain injury, potentially improving intervention strategies and clinical outcomes (22).

There have also been studies using neonatal urine proteomics to investigate peptides related to gestational age in newborns. In 2024, Maire et al. conducted proteomic analysis on the urine of 24 premature infants and 28 full-term newborns using CE-MS and LC-MS methods, identified proteins in the urine, and correlated their abundance with gestational age and clinical risk score. They confirmed a significant difference in peptide abundance between preterm and full-term infants, with SLC38A10 being one of the most abundant peptides in preterm infants. When combined with the abundance of other peptide segments, it is associated with the clinical risk score of newborns. The authors believe that further investigation of peptide clusters related to clinical outcomes can help identify premature infants with poor prognosis, thereby enabling them to benefit from early intervention (23).

There is currently limited research on these diseases using neonatal urine proteomics. However, existing studies suggest that neonatal urine proteomics holds significant potential for predicting various systemic diseases, offering promising avenues for future clinical applications.

## Summary

With the advancement of mass spectrometry, proteomics has emerged as a robust method for identifying disease biomarkers. From Electrospray Ionization Mass Spectrometry (ESI-MS) to Tandem Mass Spectrometry (MS/MS) and Liquid Chromatography-Mass Spectrometry (LC-MS), it has become highly proficient in both qualitative and quantitative protein analyses (12, 25).

Urine, a non-invasive bodily fluid, offers significant potential as a source for identifying neonatal diseases. Given that most of the protein in urine originates from the kidneys, urine offers a unique advantage in diagnosing and treating urinary system diseases. Furthermore, urine proteomics shows potential in the respiratory, digestive systems as well as nervous systems, urine proteomics could also explore the mechanism of organ development in preterm infants.

## Compared with traditional blood markers

Because urine proteomics is still in its infancy in the study of neonatal diseases, there are not many types of diseases studied, and they are all focused on small sample studies. This is an undeniable research status, but compared to traditional blood biomarker testing, this itself is developing a novel type of neonatal disease diagnosis method. Moreover, according to the papers we reviewed, urine proteomics has identified some promising biomarkers in many studies, which have statistical significance and demonstrated good predictive performance in the model. Compared with traditional blood biomarkers, biomarkers based on urine proteomics may have some advantages, such as causing pain to newborns when blood is collected, while urine based detection is non-invasive; The blood volume of newborns is limited, while urine is constantly produced; In addition, due to the ease of obtaining urine, urine based proteomic markers are more likely to be studied longitudinally at various time points, while blood markers are more difficult to achieve this. Moreover, there have been numerous studies on blood biomarkers, while research on proteomics biomarkers based on urine is not yet mature, continuing large-scale multicenter research, which may provide new insights into the pathogenesis and diagnosis of neonatal diseases.

## Qualitative and quantitative analysis of urine proteomics

Some of the studies involved in this review have been qualitative, such as the UPJO urine proteomics study, which found that some proteins are unique to newborn urine and do not exist in adult urine (11), and some proteins are specific to the urine of premature newborns in cardiovascular disease research (20). However, relatively few studies have successfully identified disease-associated protein biomarkers (15, 21), and large-scale clinical validation remains lacking. The majority of studies have focused on quantitative analysis, aiming to detect concentration differences of various proteins between disease and control groups. Based on these differential proteins, further biological function analysis or model construction is often performed. While qualitative analysis provides the foundation for quantitative research, the latter can help to validate and further explore the clinical relevance of differential proteins. With the advancement of proteomics techniques, technological progress, and large-scale studies, unique biomarkers for neonatal diseases may be identified, shedding light on more potential biomarkers relevant to neonatal health.

## Limitations and future prospects

Despite its promise, urine proteomics as a diagnostic tool for neonatal diseases faces several limitations. Firstly, there is the complexity and diversity of urine samples, for example, the collection time, collection method, processing method, storage method, and standardization of data may all affect the analysis of urine proteomics. Secondly, the inherent complexity of biological systems poses significant challenges. Thirdly, the integration and interpretation of proteomic data can be particularly difficult. Finally, the current research on neonatal urine proteomics mainly focuses on small sample studies, while there are few multi-center large sample studies and lack of clinical trial support. To address these limitations, several strategies can be implemented.

Firstly, standardizing urine collection is crucial for reliable proteomic identification. Standardized sample processing, storage methods, and data standardization are also necessary. During the sample collection phase, if the neonate can urinate autonomously, researchers can collect the urine in a clean environment. If the neonate cannot urinate, appropriate stimulation techniques can be employed to induce urination (30, 31). After urine collection, the routine procedure involves refrigerating the urine sample immediately to prevent degradation. The sample is then centrifuged at low temperature to remove cellular debris and other particulates, with the supernatant collected as the primary component for subsequent proteomic analysis. Since the protein content in urine is typically low, it may be necessary to use ultrafiltration devices or protein precipitation methods for concentration or desalting techniques to remove salts and small molecule interferents. The processed samples should then be stored at −80°C to prevent degradation. Detailed records should be kept for each urine sample processed. Quality control samples should be used for regular testing to ensure the accuracy of analytical results (14, 32). With the development of more and more neonatal urine proteomics research, it is possible to propose a universal protocol for urine proteomics detection in the future, which, combined with automated equipment, may solve this problem. Secondly, integrating multiple omics data, such as transcriptomics and metabolomics, with bioinformatics methods can enhance bioinformatics analysis. This integration allows for the exploration of molecular mechanisms underlying phenotypes and the identification of disease targets. Thirdly, employing machine learning algorithms and bioinformatics methods to annotate and model the identified biomarkers can improve the accuracy and interpretability of diagnostic models. Current research indicates that many existing models only include a small number of samples and do not distinguish between independent training and validation sets, which limits their practical application in real-world scenarios. Finally, future studies should collect more samples through large-scale, multicenter cooperation to improve the statistical efficiency of the study and enhance the external verification ability of the results.

In summary, in the future, the standardized process of urine protein detection, the integration of multiple omics data methods in urine proteomics, the interpretation of biomarker significance based on large sample urine proteomics and validation, and the development of advanced urine proteomics bioinformatics tools will all contribute to the development of neonatal urine proteomics research.

## Conclusion

The application of urinary proteomics in the field of neonatology is very limited and these studies only focused on kidney-related, respiratory-related, digestive-related, nervous systems, and organ development in preterm birth. Most of these studies are based on small samples and can only draw some preliminary conclusions. However, urinary proteomics has high potential value in exploring diagnosis and mechanism in these inspects. In the future, more urine proteomics research is needed to explore neonatal diseases and combine it with computational methods such as bioinformatics and artificial intelligence, to maximize the clinical application value of neonatal urine proteomics.
